# Modulation of posterior intestinal mucosal proteome in rainbow trout (*Oncorhynchus mykiss*) after *Yersinia ruckeri* infection

**DOI:** 10.1186/s13567-019-0673-8

**Published:** 2019-07-17

**Authors:** Gokhlesh Kumar, Karin Hummel, Ebrahim Razzazi-Fazeli, Mansour El-Matbouli

**Affiliations:** 1 Clinical Division of Fish Medicine, University of Veterinary Medicine, Vienna, Austria; 2 VetCore Facility for Research/Proteomics Unit, University of Veterinary Medicine, Vienna, Austria

## Abstract

*Yersinia ruckeri* is the causative agent of enteric redmouth disease in salmonids. In fish, the intestine represents an important site of nutrient uptake, host–pathogen interactions, and defense. The posterior intestine can be inflamed, reddened, and filled with an opaque, yellowish fluid during *Y. ruckeri* infection. Herein, we report an investigation on the proteome alteration in the posterior intestinal mucosa of rainbow trout (*Oncorhynchus mykiss*) after exposure to *Y. ruckeri*. The intestinal mucosal proteins were identified and quantified by a shotgun proteomic approach by applying data-independent quantification with sequential windowed acquisition of all theoretical mass spectra (SWATH). A total of 437 proteins were found to be differentially up- or downregulated in the posterior intestine. Gene ontology of upregulated proteins pointed to their involvement into exopeptidase, endopeptidase, and hydrolase activities, while the downregulated proteins were involved in lipid metabolism, actin binding, and translation processes. Additionally, upregulated proteins were predicted to be involved in lysosome, oxidative phosphorylation, and metabolic pathways, while downregulated proteins were implicated in focal adhesion, regulation of actin cytoskeleton, protein digestion and absorption pathways. This study showed that *Y. ruckeri* infection can alter protein abundance involved in serine-type carboxypeptidase, cysteine and aspartic-type endopeptidases, metallopeptidases, antioxidant defense, calcium ion binding, glycolytic and carbohydrate metabolic processes in the proteome of the intestinal mucosa of rainbow trout.

**Electronic supplementary material:**

The online version of this article (10.1186/s13567-019-0673-8) contains supplementary material, which is available to authorized users.

## Introduction

Enteric redmouth disease (ERM) is one of the major diseases of mainly salmonid fish. It was first described in the 1950s in rainbow trout (*Oncorhynchus mykiss*) in the Hagerman Valley of Idaho, USA. The disease is caused by *Yersinia ruckeri*, a Gram-negative rod shaped enterobacterium, and prevalent in Europe, North and South America, Australia, South Africa, the Middle East, and China [1, 2]. The signs of the disease include exophthalmia, subcutaneous hemorrhages, and splenomegaly [1]. In most cases, the intestine is inflamed, reddened, and filled with an opaque, yellowish fluid [1, 3]. In rainbow trout, up to 25% of infected fish could carry *Y. ruckeri* in their posterior intestines [4]. *Y. ruckeri* appeared to adhere or invade gills and gut tissue of rainbow trout [3, 5]. *Y. ruckeri* can also be seen in the lumen of the intestine, adhering to mucus and invading villi within 30 min after exposure and can be detected 7 days post-exposure (dpe) in the liver, spleen, brain, and heart [6].

The strains of *Y. ruckeri* have been classified on the basis of biotypes, serotypes (O1, O2, O3, and O4), and outer-membrane protein types [7]. The majority of epizootics in salmonids are caused by the motile serotype O1a [1]. There are two biotypes of *Y. ruckeri;* biotype 1 is positive for motility and lipase secretion, whereas biotype 2 is negative for both tests [8]. The outbreaks of ERM were recorded in biotype 1-vaccinated salmonids and the isolates associated with these outbreaks were identified as *Y. ruckeri* biotype 2 [9–12].

In fish, the gastrointestinal (GI) tract is a multifunctional organ, which serves a diverse range of functions from nutrient absorption to ionic and osmotic regulation and even air breathing [13–19]. Gut epithelial cells are protected by a mucus layer, which creates a physical and chemical barrier against an intruder and acts as an important mechanism of innate defense that maintains tissue homeostasis [20]. The GI tract is continuously challenged with food antigens as well as pathogens entering the body via feed and water intake, and acts as the first line of defense against pathogen attachment and invasion [20–22]. The posterior portion of the GI tract is a site where uptake of macromolecules and foreign antigens occurs, which leads to antigen uptake and processing as well as the initiation of a systemic immune response [20, 23]. The disease has been of a major concern in salmonids, which influences the physiological response of fish. Rainbow trout is highly susceptible to ERM [1]. The effect of natural herbal products on growth performance, hematological and biochemical values and resistance to *Y. ruckeri* infection was studied as a feed additives for rainbow trout [24, 25].

In ERM, hyperemia and hemorrhages throughout the intestinal mucosa in rainbow trout have been observed [1]. The intestinal response of rainbow trout has been mainly studied at the transcription level, especially regarding immune gene expression [26–29]. It is still largely unknown how the gut mucosal proteome of the rainbow trout responds to alternations in the luminal environment triggered by *Y. ruckeri.* Therefore, in the current study, we aimed to evaluate the possible effects of *Y. ruckeri* exposure on the modulation of the proteome profile in the posterior intestine of rainbow trout. In order to meet this aim, we applied a label-free shotgun proteomic approach.

## Materials and methods

### Collecting posterior intestine samples

The details of the experimental setup have been described in our previous study [30]. Briefly, specific pathogen free rainbow trout (15 ± 1 cm) were allocated to 9 aquaria, 18 fish per aquarium. In order to monitor the effect of *Y. ruckeri* exposure, the following three treatments were given in parallel—Treatment 1: an exposure with biotype 1 (CSF007–82), Treatment 2: an exposure with biotype 2 (A7959–11), and Treatment 3: a mock exposure to sterile tryptic soya broth. In both the test groups, the fish were challenged by bath exposing to 2 × 10^6^ colony forming units (CFU) of *Y. ruckeri* biotype 1 (CSF007-82) and *Y. ruckeri* biotype 2 (A7959-11) strains for 2 h. The fish were maintained in a flow-through system supplied with UV-treated ground water at 19 ± 1 °C, monitored daily, and morbid, moribund, and dead fish were taken out immediately from the aquaria. The mortality of fish was only considered to be caused by *Y. ruckeri* if the bacteria were recovered from the head kidney and confirmed using the MONO-Yr kit (Bionor, Skien, Norway) or PCR [31]. No morbid and moribund fish were sampled for the study. The fish were maintained in starvation 48 h before the sampling. Nine fish from each group were sampled at 3, 9, and 28 dpe. The posterior intestine of each sample was dissected and placed in cold sterile PBS (Sigma-Aldrich, Neustadt, Germany). The intestine was opened along the mesenteric border and washed three times with cold sterile PBS containing a cocktail of mammalian protease inhibitors (Sigma-Aldrich) to remove digesta and fecal matter. Intestinal mucosa was scraped with a sterile large scalpel blade. Each sample was divided into two parts, one immediately snap-frozen in liquid nitrogen for proteomic analysis, and one fixed in RNA*Later* (Sigma-Aldrich) for molecular analysis and stored at −80 °C.

### Protein extraction

Equal amounts (30 mg) of nine individual intestinal mucosal samples from each group were pooled randomly as three pools of three samples each in order to minimize the effects of individual variation. It refers to *N* = 3 for the biological replicates per time point (3, 9 and 28 dpe) and per exposed group (control, *Y. ruckeri* biotype 1 and biotype 2). Each pool was ground using a sterilized mortar and pestle in the presence of liquid nitrogen to a fine powder, which was then mixed with 800 μL of precooled denaturing lysis buffer (7 M urea, 2 M thiourea, 4% CHAPS, and 1% DTT) containing mammalian protease inhibitor cocktail (Sigma-Aldrich). The samples were subjected to ultrasonic disruption for 10 cycles of 10 s pulse-on and 30 s pulse-off. The samples were then centrifuged at 12 000 rpm for 20 min at 4 °C to remove any cellular debris. The supernatant was collected. The protein content was measured using the Pierce 660 nm Protein Assay (Thermo Scientific, Vienna, Austria).

### Protein digestion

The protein digestion was performed following a standard enhanced, filter-aided sample preparation protocol (FASP) [32]. Briefly, after washing, proteins were reduced and alkylated. On-filter digestion was performed with 1.2 µg Trypsin/Lys-C mix (Promega, Madison, USA) for 14 h at 37 °C. Digested peptides were recovered, dried, and redissolved in 0.1% aqueous trifluoroacetic acid. After desalting and sample cleanup by C18 ZipTips (Sigma-Aldrich), all samples were spiked with standardized indexed retention time reference peptides for facilitation of retention time alignment (iRT kit, Biognosys, Switzerland). For each liquid chromatography (LC)–mass spectrometer (MS) analysis, 3 µg of digested protein was loaded onto the LC column.

## Microflow high-performance liquid chromatography electrospray-ionisation quadrupole time-of-flight tandem mass spectrometry (micro LC ESI QTOF MS/MS)

Micro LC ESI QTOF MS/MS was carried out as described earlier [30]. Briefly, peptides were separated on an Eksigent NanoLC 425 system using a microflow pump module (Sciex, Concord, Canada). The samples were pre-concentrated and desalted on a 5 mm YMC-Triart C18 precolumn using ultra-pure LC–MS grade water with 0.1% formic acid as a mobile phase and a flow rate of 10 μL/min. Desalted peptides were separated on a 15 cm YMC-Triart C18 column at a flow rate of 5 µL/min. Mobile phase A was ultra-pure water with 0.1% formic acid, whereas mobile phase B consisted of acetonitrile with 0.1% formic acid. The gradient started with 3% B and increased in two steps to 25% B (68 min) and 35% (73 min) followed by a washing step with 80% B. Total acquisition time was 87 min. For mass spectrometric analysis, LC was directly coupled via a DuoSpray ion source in electrospray mode to a high resolution quadrupole time-of-flight mass spectrometer (Triple TOF 5600+, Sciex). For information dependent data acquisition (IDA runs), the MS1 spectra were collected in a range of 400–1250 m/z for 250 ms. The 40 most intense precursors with charge state 2–4, which exceeded 150 counts per second, were selected for fragmentation. MS2 spectra were collected in the range of 200–1500 m/z for 50 ms. The precursor ions were dynamically excluded from reselection for 13 s. Based on the data-dependant acquisition spectra, an ion library was established for the next step of data-independent acquisition of SWATH spectra (Sequential Windowed Acquisition of All Theoretical Mass Spectra). Seventy-three variable windows were created in a mass range of 400–1250 Da depending on the precursor ion density. All precursors of each window were fragmented. MS2 spectra were acquired for 50 ms (SWATH runs).

### Data processing

The acquired raw data were processed with ProteinPilot Software version 5.0 (Sciex) for re-calibration and database searches as described by Kumar et al. [30]. The database contained entries of following taxonomies: *Oncorhynchus mykiss* (NCBI, Refseq: 71 285 entries), and *Y. ruckeri* (UniProt, taxonomy id 29486: 4493 entries) as well as cRAP (common Repository of Adventitious Proteins). The database search parameters applied were trypsin digestion, cysteine alkylation set to iodoacetamide, search effort set to rapid ID. The mass tolerance in MS mode was set by program default with 0.05 Da in MS and 0.1 Da in MSMS mode for the rapid recalibration search, and 0.0011 Da in MS and 0.01 Da in MSMS mode for the final search. False discovery rate (FDR) analysis was performed using the integrated tools in ProteinPilot with < 1% on peptide as well as on protein level.

For the quantification of proteins by SWATH, the identified proteins of a combined search of all 27 IDA runs identifying 3425 proteins served as a basis for the creation of the SWATH ion library with the MS/MS (ALL) with SWATH Acquisition MicroApp 2.0 in PeakView 2.2 (both Sciex) (dataset PRIDE PXD011087, IDA FASP intestine Control/CSF/7959). Shared peptides were excluded. So the resulting ion library (Additional file 1) contained a total of 3372 proteins, 25 469 peptides and 295 168 transitions to be quantified potentially. The key criteria for processing of the SWATH samples were to use only proteins with an FDR rate below 1%, furthermore up to 6 peptides per protein and up to 6 transitions per peptide were chosen, false discovery rate threshold was 5%, peptide confidence threshold 98%, XIC extraction window 5 min, XIC width 100 ppm and modified peptides were excluded. Retention time alignment based on iRT peptides (iRT kit, Biognosys) and processing of SWATH raw samples for calculation of raw peak areas were performed in PeakView 2.2. These were normalized in the software MarkerView 1.2.1 (Sciex) with the integrated tool based on the total area sums, which assumes that the abundance of most of the proteins within the label free quantification approach remains unchanged and only a few of them are differentially regulated.

### Data analysis

All statistical analyses were performed in R programming language [33]. The protein abundance after retention time alignment and normalization on total area sums derived from MarkerView were first transformed into a logarithmic scale. Technical replicates compensating for instabilities during LC–MS acquisition (*N* = 2) were averaged for the mean values. Afterward, the differences in abundance of posterior intestinal proteins were assessed using one-way ANOVA for each protein comparing groups [(control vs. biotype 1 (CSF007-82) exposed vs. biotype 2 (A7959-11) exposed posterior intestine samples] for each time point (day 3, 9 and 28). The method of Benjamini and Hochberg [34] was applied to control the FDR. The differences between the biotypes-exposed and control posterior intestine samples were considered significant if FDR-adjusted *p*-values were smaller than the significance level of *α* = 0.05. In order to assess the significance of the pairwise comparisons, Tukey’s honest significant difference method was applied as a post hoc test. Protein level changes were considered differential if the adjusted *p* value was below *α* and the absolute fold change was at least two (fold change < −2 or > +2).

### GO annotation

PANTHER classification system was used to categorize the Gene Ontology of all the differentially up- or downregulated proteins in biological process, cellular component, and protein class [35]. Molecular function and Kyoto Encyclopedia of Genes and Genomes (KEGG) pathway of differentially up- or downregulated proteins were further determined on the Cytoscape software (version 3.7.1) [36] plugin ClueGO (version 2.5.4) [37]. We tested the significance of GO term and KEGG pathway using Fisher’s exact test and FDR-correction at< 0.05 significant level.

### Quantitative real time PCR

In order to validate the abundance of identified proteins, eight kinds of proteins (probable serine carboxypeptidase, cathepsin D, caspase 6, lysozyme C II, precerebellin, protein S100, and tubulin alpha) were chosen for qPCR analysis carried out on a CFX96 Touch Real-Time PCR detection system (Bio-Rad, Hercules, USA). The sequences of the primers used in this study are listed in Additional file 2. Total RNA was extracted from posterior intestinal mucosa samples using an RNeasy Mini Kit (Qiagen, Hilden, Germany) and included an on-column DNase digestion step. cDNA was synthesized using an iScript cDNA Synthesis Kit (Bio-Rad) with 500 ng total RNA according to the user’s manual.

All reactions were done in a final volume of 20 μL, which contained 4 μL of 1:10-fold diluted cDNA, 0.5 μL of each primer (10 pmol/μL), 10 μL of 2× SsoAdvanced™ Universal SYBR Green Supermix (Bio-Rad), and 5 μL of RNase-free water. After 5 min of cDNA denaturation at 95 °C, 38 cycles were performed at 95 °C for 30 s, 57 °C for 30 s and 72 °C for 30 s. Each qPCR was performed for all biological replicates. Relative gene expression was assessed by the CFX Manager Maestro Software in normalized expression mode (∆∆Cq), using elongation factor alpha 1 as a Ref. [38], and was compared to the control sample. Additionally, standard curve was generated for qPCR using plasmid containing *Y. ruckeri* 16S ribosomal RNA fragment [39]. *Y. ruckeri* load was measured in the exposed and unexposed control posterior intestine samples with a *Y. ruckeri* 16S rRNA specific primers [39]. The copy number of *Y. ruckeri* 16S rRNA was calculated for each sample. The differences between unexposed control and exposed group and between exposed groups were tested for significant differences using a *t*-test. The sequential differences within exposed groups were tested for using a one-way ANOVA and significant differences revealed with the Tukey’s post hoc test. The Pearson correlation coefficient (*r*) was calculated to estimate the relationship between qPCR and proteomic quantifications. For all statistical tests, a *p*-value of < 0.05 was regarded as significant and all the data were analyzed in IBM SPSS software version 24.

## Results

### Clinical signs and *Y. ruckeri* load

**Figure 1:**
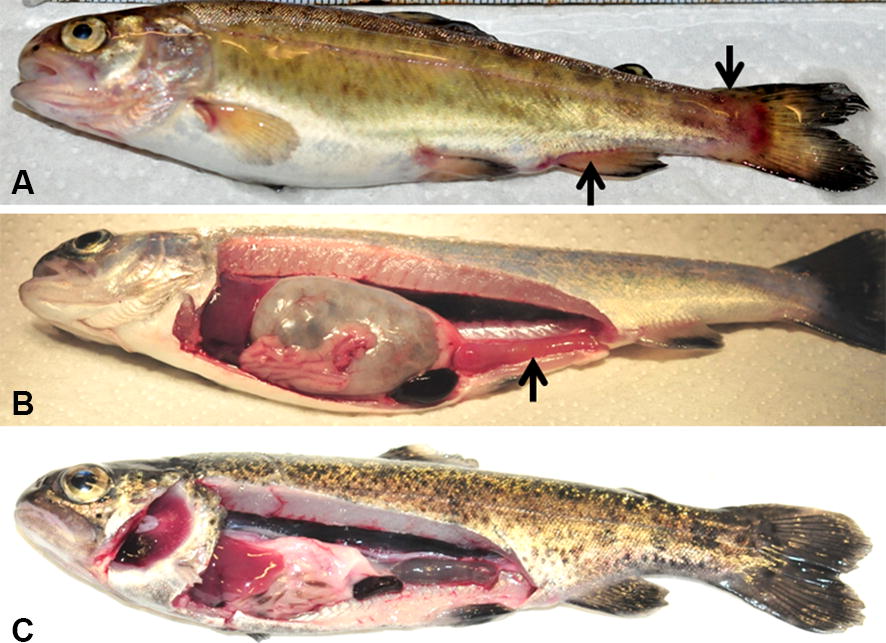
**Rainbow trout showing clinical signs of enteric redmouth disease. A** Hemorrhages in the caudal and anal fins (arrows). **B** Enlarged spleen and reddened intestine (arrow), and **C** rainbow trout at 28 days post-exposure.

**Figure 2:**
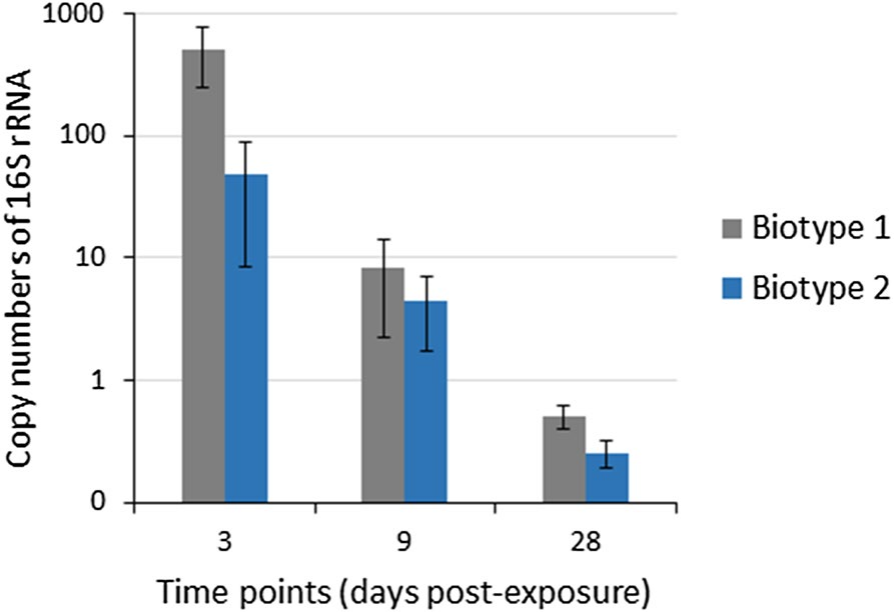
***Yersinia ruckeri*** **16S rRNA copy numbers in the posterior intestine of rainbow trout.** Quantitative real-time PCR shows the mean copy numbers of 16S rRNA gene in the exposed posterior intestine samples. There were significant differences of copy numbers of *Y. ruckeri* between time points (*p* value = 0.001), and between biotype 1 and biotype 2 (*p* value = 0.01). No *Y. ruckeri* was detected in unexposed control posterior intestine samples at any time point. Error bars indicate standard deviation (*n* = 9).

The *Y. ruckeri*-exposed fish were prepared as described in our previous study [30], where 30% fish showed severe clinical signs of the disease and the maximum morbidity and mortality were observed between 8 and 10 dpe. The fish exhibited external hemorrhages in the caudal and anal fins (Figure 1A) and internal signs, such as enlarged spleen and reddened intestine (Figure 1B), at 9 dpe. However, at 28 dpe, these organs were in normal form and did not show any clinical signs of the disease (Figure 1C). The *Y. ruckeri* 16S transcript increased and peaked in the fish exposed to both biotypes at 3 dpe and then decreased at 9 dpe and was almost undetected at 28 dpe (Figure 2). No *Y. ruckeri* was detected in unexposed control samples.

### Protein quantification

A total of 3006 proteins in the posterior intestine of rainbow trout were identified at 1% FDR and minimum of two matching peptides (Additional file 3) over all 27 samples including controls as well as the exposed groups. In total, 1880 proteins with at least two unique peptides could be quantified. Sixty-two percent of the total 3006 proteins identified amongst all samples could be quantified, after the exclusion of shared and modified peptides and the exclusion of so-called one-peptide wonders. The statistical analysis (exposed versus control) revealed a total of 437 differentially abundant proteins in the posterior intestine at 3 dpe (Additional file 4). Of these, 205 proteins were upregulated and 232 proteins were downregulated in the posterior intestine at 3 dpe. We did not find statistically different changes in the protein abundance at 9 and 28 dpe. As shown in Table 1, upregulated proteins were related to serine -type carboxypeptidase, cysteine-type endopeptidase, aspartic-type endopeptidase, metallodipeptidase, metallopeptidase, hydrolase, peroxidase activity, signal transduction, and carbohydrate metabolic activities. On the other hand, the downregulated proteins were related to lipid metabolism, actin filament binding, stress response, and translation (Table 2).

**Table 1:** **List of upregulated posterior intestine proteins of rainbow trout in response to** ***Yersinia ruckeri*** **strains**

Accession Refseq	Protein name	Number of quantified peptides	Biological process	Intestine in response to *Y. ruckeri* strain	3 dpe (fold change)	9 dpe (fold change)	28 dpe (fold change)
XP_021441860.1	Probable serine carboxypeptidase CPVL	6	Serine-type carboxypeptidase activity	Biotype 1	3.6*	−1.7	1.4
				Biotype 2	4.9*	−1.3	1.4
XP_021462823.1	Dipeptidase 1	6	Metallodipeptidase activity	Biotype 1	2.1*	1.3	−1.0
				Biotype 2	2.7*	1.5	−1.2
XP_021442206.1	Xaa-Pro dipeptidase	6	Metallopeptidase activity	Biotype 1	2.4*	−1.1	1.2
				Biotype 2	3.1*	1.1	1.2
XP_021437213.1	Dipeptidyl peptidase 1	5	Cysteine-type endopeptidase activity	Biotype 1	1.8	1.2	1.9
				Biotype 2	2.3*	1.3	1.5
XP_021460613.1	Cathepsin D isoform X1	6	Aspartic-type endopeptidase activity	Biotype 1	2.4*	−1.1	1.2
				Biotype 2	3.2*	1.1	1.2
XP_021427100.1	Pro-cathepsin H	5	Aminopeptidase activity	Biotype 1	4.3*	−2.0	1.3
				Biotype 2	6.3*	−1.3	1.0
XP_021430445.1	Cathepsin L1	6	Cysteine-type endopeptidase activity	Biotype 1	4.3*	−2.4	1.3
				Biotype 2	5.9*	−1.4	1.1
XP_021430112.1	Acid phosphatase type 7	6	Acid phosphatase activity	Biotype 1	2.5*	1.1	1.8
				Biotype 2	4.4*	1.7	1.3
XP_021457257.1	Lysozyme C II	5	Hydrolase activity	Biotype 1	2.6*	2.2*	1.4
				Biotype 2	2.8*	1.6	1.1
XP_021478264.1	Macrophage mannose receptor 1	5	Endocytosis	Biotype 1	3.2*	−1.1	1.3
				Biotype 2	4.7*	1.4	1.2
XP_021446773.1	Eosinophil peroxidase	6	Peroxidase activity	Biotype 1	3.2*	−1.2	−1.1
				Biotype 2	1.6	−1.4	−1.2
XP_021424232.1	Thioredoxin reductase 1	4	Redox homeostasis	Biotype 1	1.7	1.1	1.1
				Biotype 2	2.2*	1.0	1.1
XP_021412083.1	Lysosomal alpha-mannosidase	6	Alpha-mannosidase activity	Biotype 1	2.8*	−1.8	1.4
				Biotype 2	4.8*	1.1	1.3
NP_001154031.1	Glutathione S-transferase A	6	Glutathione metabolic process	Biotype 1	1.7	1.1	−1.0
				Biotype 2	2.0*	1.2	−1.0
XP_021460921.1	Peroxisomal multifunctional enzyme type 2	6	Oxidoreductase activity	Biotype 1	2.0*	1.1	−1.0
				Biotype 2	2.4*	1.0	−1.0
XP_021475660.1	Endoplasmic reticulum aminopeptidase 1	4	Aminopeptidase activity	Biotype 1	1.8	1.2	1.0
				Biotype 2	2.2*	1.0	−1.0
NP_001117743.1	Caspase 6 precursor	2	Apoptotic process	Biotype 1	2.2*	−1.1	1.2
				Biotype 2	2.1*	−1.2	−1.1
XP_021477916.1	Interferon-induced GTP-binding protein Mx2	5	GTPase activity	Biotype 1	2.0	1.3	−1.1
				Biotype 2	2.4*	1.3	−1.3
XP_021468639.1	Gamma-interferon-inducible lysosomal thiol reductase	3	Antigen processing and presentation	Biotype 1	3.0*	−1.9	1.4
				Biotype 2	4.4*	−1.1	1.3
XP_021466891.1	Precerebellin-like protein	5	Response to lipopolysaccharide	Biotype 1	2.9*	5.3*	2.0
				Biotype 2	2.2	3.3*	1.3
XP_021453615.1	Calretinin-like	6	Calcium ion binding	Biotype 1	2.5*	1.5	1.2
				Biotype 2	3.0*	1.6	1.0
XP_021426220.1	Regucalcin	6	Calcium ion binding	Biotype 1	2.1	1.0	1.4
				Biotype 2	3.1*	1.2	1.1
XP_021480508.1	Ras-related protein Rab-5C	4	GTPase activity	Biotype 1	1.7	1.0	1.3
				Biotype 2	2.4*	1.1	1.0
XP_021477941.1	Fructose-1,6-bisphosphatase 1	3	Carbohydrate metabolic process	Biotype 1	3.8*	1.4	1.0
				Biotype 2	2.2	1.0	−1.2
XP_021434786.1	Beta-hexosaminidase	2	Carbohydrate metabolic process	Biotype 1	2.4*	−1.5	−1.0
				Biotype 2	2.5*	−1.1	1.2
XP_021467816.1	Alpha-*N*-acetylgalactosaminidase	6	Carbohydrate metabolic process	Biotype 1	3.0*	−1.3	1.2
				Biotype 2	4.1*	1.1	1.3

Fold change was statistically analyzed in the posterior intestine of rainbow trout exposed to *Y. ruckeri* biotype 1 (CSF007-82) and biotype 2 (A7959-11) versus control posterior intestine of rainbow trout samples (*n* = 3 per time point).

* Statistically significant difference according to both ANOVA and post hoc Tukey’s HSD with FDR-adjusted *p*-value < 0.05 and fold change < −2 or > +2. (Full table is presented in Additional file 4).

**Table 2:** **List of downregulated posterior intestine proteins of rainbow trout in response to** ***Yersinia ruckeri*** **strains**

Accession Refseq	Protein name	Number of quantified peptides	Biological process	Intestine in response to *Y. ruckeri* strain	3 dpe (fold change)	9 dpe (fold change)	28 dpe (fold change)
NP_001117719.1	Apolipoprotein A-I-1	6	Lipid metabolism	Biotype 1	−1.3	−1.1	−1.6
				Biotype 2	−2.2*	−1.2	−1.1
NP_001154920.1	Apolipoprotein A-II	4	Lipid metabolism	Biotype 1	−2.1*	−1.0	−1.7
				Biotype 2	−4.0*	1.1	−1.3
XP_021447265.1	Apolipoprotein C-I	2	Lipid metabolism	Biotype 1	−1.7	1.1	−3.1*
				Biotype 2	−4.6*	−1.3	−2.7
XP_021422847.1	Filamin-A	6	Actin filament binding	Biotype 1	−3.2*	−2.8	−1.5
				Biotype 2	−10.4*	−2.8	−1.4
XP_021438757.1	Alpha-actinin-1	6	Actin filament binding	Biotype 1	−2.4*	−1.7	−1.2
				Biotype 2	−6.3*	−1.9	1.3
XP_021474949.1	PDZ and LIM domain protein 3	2	Alpha-actinin binding	Biotype 1	−4.2*	−2.5*	−1.1
				Biotype 2	−22.6*	−1.7	1.1
XP_021479779.1	Dihydropyrimidinase-related protein 3	6	Actin filament bundle assembly	Biotype 1	−2.4*	−1.7	−1.3
				Biotype 2	−4.5*	−1.6	−1.1
XP_021449109.1	Tubulin alpha chain	3	Microtubule process	Biotype 1	−2.1*	1.2	−1.8
				Biotype 2	−4.9*	−1.2	−1.6
XP_021451765.1	Tubulin polymerization-promoting protein	6	Microtubule binding	Biotype 1	−3.6*	−1.7	−1.3
				Biotype 2	−7.0*	−1.5	−1.2
XP_021480500.1	DnaJ homolog subfamily C member 7	2	Chaperone binding	Biotype 1	−1.9	1.1	−1.2
				Biotype 2	−3.2*	−1.4	−1.0
XP_021478259.1	Lysosome membrane protein 2	2	Chaperone activity	Biotype 1	−2.6*	1.1	−1.3
				Biotype 2	−3.3*	−1.3	−1.3
NP_001117706.1	Heat shock 47 precursor	6	Stress response	Biotype 1	−3.7*	−1.4	−1.1
				Biotype 2	−9.6*	−1.2	1.0
XP_021438340.1	Heat shock cognate 70 kDa protein-like	6	Chaperone activity	Biotype 1	−1.9	−1.1	−1.3
				Biotype 2	−2.6*	−1.4	−1.1
XP_021441996.1	Programmed cell death protein 5	2	DNA binding	Biotype 1	−2.7*	1.2	−1.7
				Biotype 2	−4.4*	−1.4	−2.6*
XP_021418468.1	Annexin A13	6	Calcium ion binding	Biotype 1	−2.0*	−1.6	−1.8
				Biotype 2	−3.4*	−1.2	−1.1
XP_021416101.1	Protein S100 A13	2	Calcium binding	Biotype 1	−3.2*	−1.1	−1.5
				Biotype 2	−6.6*	−1.3	−1.1
NP_001117701.1	M-calpain	6	Calcium ion binding	Biotype 1	−2.0*	−1.2	−1.0
				Biotype 2	−4.0*	−1.4	1.0
NP_001117963.1	Calpain 2 catalytic subunit	5	Calcium ion binding	Biotype 1	−1.8	−1.1	−1.1
				Biotype 2	−2.6*	−1.0	−1.0
XP_021419099.1	l-Lactate dehydrogenase	2	Carboxylic acid metabolic process	Biotype 1	−3.4*	−1.5	−1.8
				Biotype 2	−7.4*	−1.7	1.3
XP_021452473.1	Glyceraldehyde-3-phosphate dehydrogenase	4	Glycolysis process	Biotype 1	−1.8	−1.2	−1.3
				Biotype 2	−2.9*	−1.1	−1.1
XP_021447060.1	Pyruvate dehydrogenase E1	4	Glucose metabolic process	Biotype 1	−2.1*	1.2	1.0
				Biotype 2	−1.7	1.1	−1.0
XP_021440913.1	Galectin-5-like isoform X2	2	Carbohydrate binding	Biotype 1	−2.3*	1.0	1.1
				Biotype 2	−3.9*	1.4	−1.2
XP_021476056.1	Eukaryotic translation initiation factor 5A	5	Translation	Biotype 1	−1.4	1.1	−1.4
				Biotype 2	−2.4*	−1.3	−1.6
XP_021467048.1	40S ribosomal protein S12	5	Translation	Biotype 1	−1.1	1.4	−1.2
				Biotype 2	−2.2*	1.2	1.3
XP_021456583.1	40S ribosomal protein S29	3	Translation	Biotype 1	−3.4*	1.0	−1.2
				Biotype 2	−2.9*	−1.0	−1.3

Fold change was statistically analyzed in the posterior intestine of rainbow trout exposed to *Y. ruckeri* biotype 1 (CSF007-82) and biotype 2 (A7959-11) versus control posterior intestine of rainbow trout samples (*n* = 3 per time point).

* Statistically significant difference according to both ANOVA and post hoc Tukey’s HSD with FDR-adjusted *p*-value < 0.05 and fold change < −2 or > +2. (Full table is presented in Additional file 4).

### GO annotation

**Figure 3:**
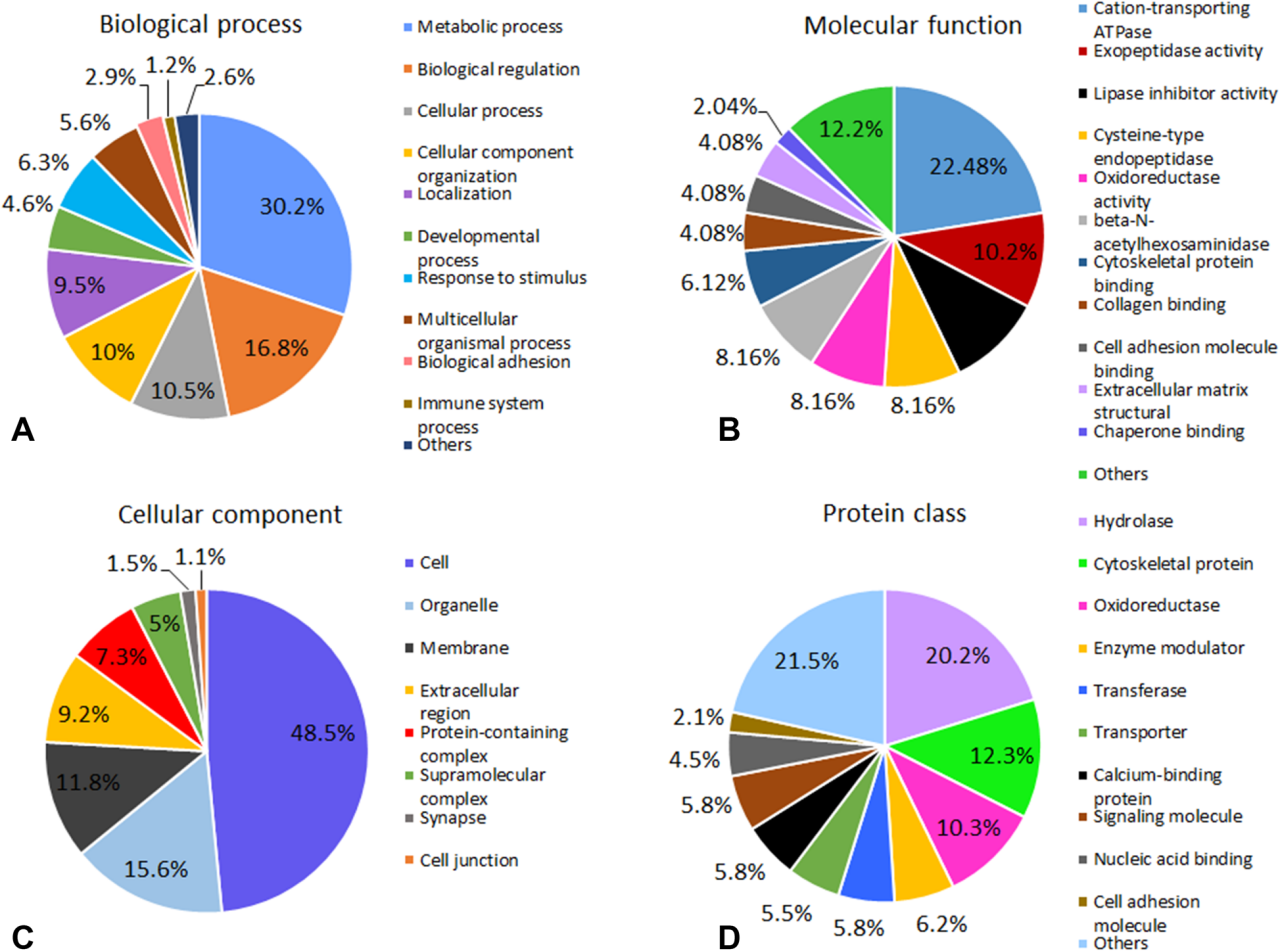
**Classification of differentially up or down-regulated posterior intestine proteins of rainbow trout in response to** ***Yersinia ruckeri*** **at 3 dpe.** Proteins were classified by gene ontology terms for biological processes, molecular functions, cellular components and protein classes using PANTHER and ClueGO tools. **A** Biological process, **B** molecular function, **C** cellular component, and **D** protein class.

Within the classification of biological process, the differentially up- or downregulated posterior intestine proteins were mainly associated with metabolic process, biological regulation, cellular processes, and cellular component organization (Figure 3A). In terms of molecular function, the majorities of up- or downregulated posterior intestine proteins were involved in cation-transporting ATPase, exopeptidase, lipase inhibitor, cysteine-type endopeptidase, oxidoreductase, and beta-*N*-acetylhexosaminidase activities (Figure 3B). Most of the proteins were localized in the cell, organelle, membrane, and extracellular region (Figure 3C). Classification based on protein class resulted in the identification of ten major categories: hydrolase, cytoskeletal protein, oxidoreductase, enzyme modulator, transferase, transporter, calcium binding protein, signaling molecule, nucleic acid binding, and cell adhesion molecule (Figure 3D). Additionally, upregulated proteins were predicted to be involved in lysosome, oxidative phosphorylation, and metabolic pathways, while downregulated proteins were involved in focal adhesion, regulation of actin cytoskeleton, protein digestion and absorption pathways.

### Validation of differentially abundant proteins

**Figure 4:**
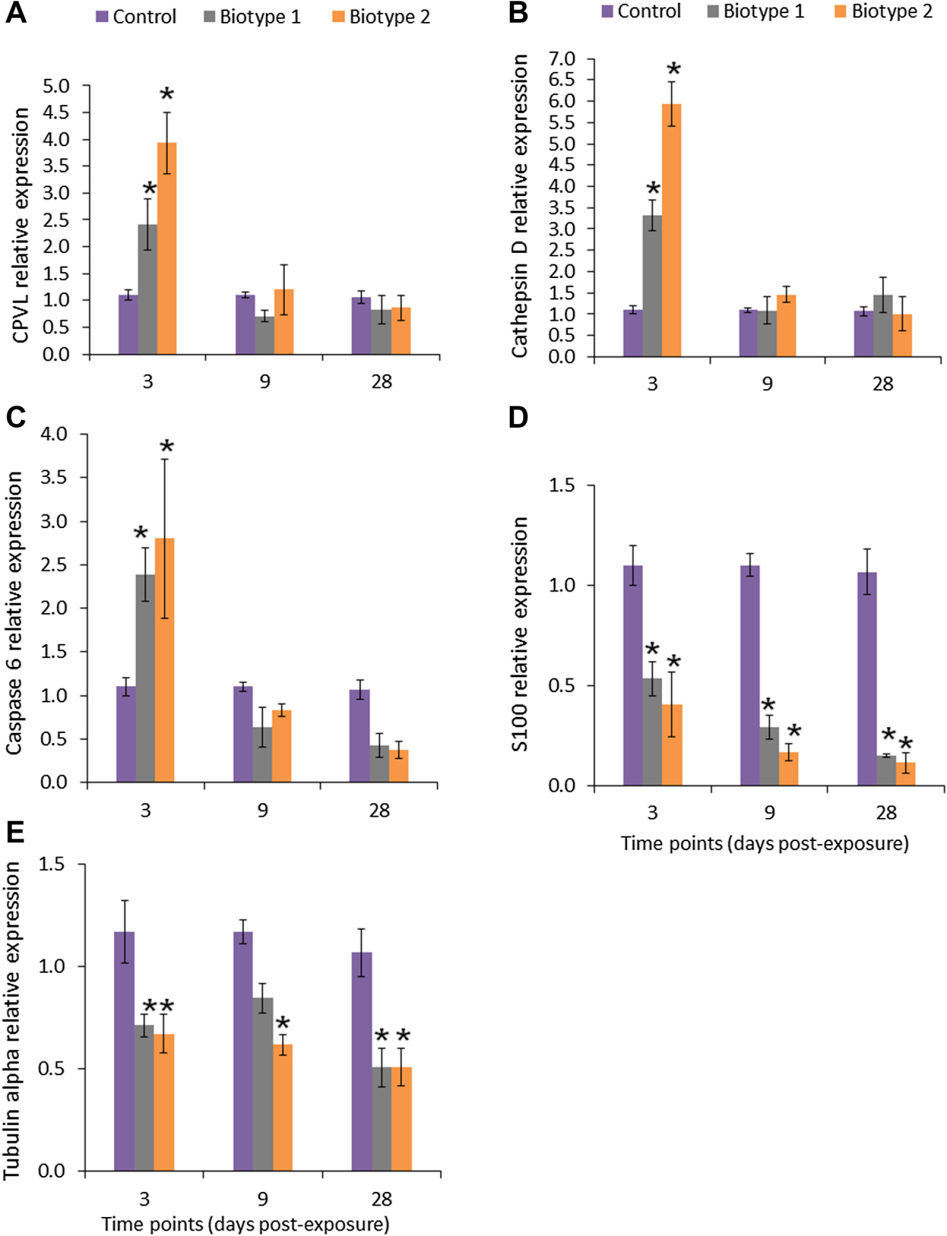
**Relative expression levels of probable serine carboxypeptidase, cathepsin D, caspase-6, protein S100 and tubulin alpha in the posterior intestine of rainbow trout.** Quantitative real-time PCR shows mean relative expression profiles of each selected gene in the posterior intestine of rainbow trout in response to *Y. ruckeri* biotype 1 (CSF007-82) and biotype 2 (A7959-11) at different time points. Relative gene expression changes in each gene were determined in the exposed and control posterior intestine samples by the CFX Manager Maestro Software in normalized expression mode (∆∆Cq), using elongation factor alpha 1 as a reference at each time point. **A** Probable serine carboxypeptidase CPVL, **B** cathepsin D, **C** caspase 6, **D** protein S100, and **E** tubulin alpha. Stars indicate statistically significant differences in the gene expression compared to the control group. Error bars indicate standard deviation (*n* = 9).

**Figure 5:**
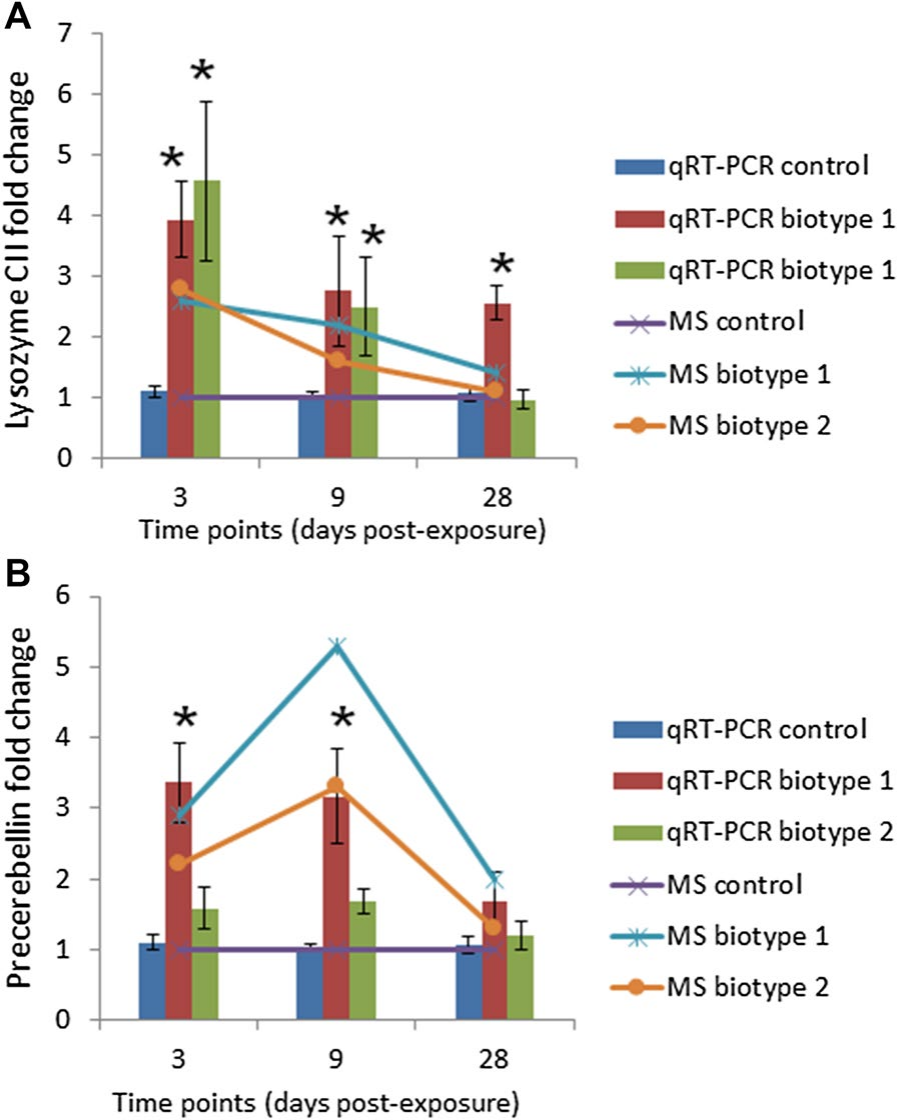
**Comparison of transcriptional analysis and MS-based proteomic results of lysozyme C II and precerebellin.** The qRT-PCR and the MS-based proteomic results are presented by the bar and line charts, respectively. Stars indicate statistically significant differences in the fold change compared to the control group. Error bars indicate standard deviation (*n* = 9). **A** Lysozyme C II, and **B** precerebellin-like protein.

The upregulation of six proteins (probable serine carboxypeptidase, cathepsin D, caspase 6, lysozyme C II, and precerebellin-like protein) and downregulation of two proteins (protein S100 and tubulin alpha) in the posterior intestine of the fish exposed to both biotypes were confirmed at the mRNA level by qPCR analysis. The transcript levels of these candidate genes were significantly (*p* < 0.05) either increased or decreased compared to the control posterior intestine samples (Figure 4). The results of qPCR were consistent with those obtained from the proteomic results mainly at 3 dpe (Figure 5), which confirmed reliability of our proteomic data (*r* = 0.406 or 0.877). Nevertheless, the transcript expression slightly varied in parallel to the corresponding protein abundance perhaps suggesting additional post-transcriptional regulation.

## Discussion

The gut immune system of teleost fish deserves special attention in the biology of the fish intestine. This provides important insights into the mechanisms induced by the progression of diseases [14]. In the case of common carp (*Cyprinus carpio*), the response of intestinal mucosa against *Aeromonas hydrophila* was investigated at the proteome level [40], which improved the understanding of defense mechanisms of carp intestinal mucosa and associated molecular mechanisms. In rainbow trout, the intestinal response was monitored at transcription levels against pathogens like *Y. ruckeri*, *Enteromyxum leei*, *Edwardsiella ictaluri*, and *Vibrio harveyi* [26–29]. In the current study, the elements of the gut mucosal responses were examined by proteome profiling of the posterior intestine of rainbow trout following experimental exposure to *Y. ruckeri*. Additionally, proteins were significantly up- or downregulated in the posterior intestine mainly at 3 dpe, which correlated with increasing bacterial transcripts found in the posterior intestine at 3 dpe (Figure 2). This suggests that *Y. ruckeri* might affect the gut and modulate the intestinal proteome of fish whenever a high load of bacteria is present in the fish intestine.

In total, 30% mortality was found in rainbow trout following the exposure to *Y. ruckeri* (2 × 10^6^ CFU) [30]. This mortality rate is in contrast with the previous studies [41, 42], where mortality in rainbow trout was observed to be 20% and 66%, respectively, at lower (5 × 10^5^ CFU) and higher (1.8 × 10^9^ CFU) levels of the challenge dose of *Y. ruckeri*. These differences in the mortality data may be due to variations between the bacterial strains, challenge doses, fish size, and laboratory conditions. At 28 dpe, the intestine of rainbow trout exposed to *Y. ruckeri* strains was in normal form and also transcripts of *Y. ruckeri* 16S were almost undetectable in the posterior intestine. It seems that rainbow trout slowly cleared the *Y. ruckeri* during the analyzed period.

We found upregulation of exopeptidase and endopeptidase [probable serine carboxypeptidase (3.4- to 4.7-fold), dipeptidase I (2.1- to 2.7-fold), Xaa-Pro dipeptidase (2.4- to 3.1-fold), dipeptidyl peptidase 1 (2.3-fold), cathepsin D (2.4- to 3.2-fold), pro-cathepsin H (4.3- to 6.3-fold) and cathepsin L1 (4.3- to 5.9-fold)] in the posterior intestine of rainbow trout exposed to *Y. ruckeri*. These exo- and endopeptidases participate in several physiological and cellular processes in the guts of animals [43]. The expression of these peptidase (dipeptidase I and cathepsin D) genes was observed in the gut of channel catfish (*Ictalurus punctatus*) and grass carp (*Ctenopharyngodon idella*) following *Edwardsiella ictaluri* and *Aeromonas hydrophila* challenges [44, 45], thereby suggesting that they participate in various degradation functions and digestive processes and are closely involved in the immune response of rainbow trout. Overall, our study suggests that proteolysis and peptide hydrolysis activities are upregulated in the GI tract during the bacterial infection, indicating that these identified peptidases might play some role in host defense.

Furthermore, we found upregulation of phagocytosis proteins such as lysozyme C II, macrophage mannose receptor 1, eosinophil peroxidase, and thioredoxin reductase 1 in the posterior intestine of rainbow trout. These phagocytosis proteins were also upregulated in head kidney and spleen of rainbow trout exposed to *Y. ruckeri* strains [30]. Lysozymes are important defense proteins of the innate immune system of fish against bacterial pathogens [24, 46]. The upregulation of lysozyme C II (3.3-fold) in the posterior intestine, suggests that, besides its role in defense against bacterial pathogens, this protein may also play a significant role in the digestion during the infection in fish. In addition, the upregulation of macrophage mannose receptor 1 (3.2- to 4.7-fold) and thioredoxin reductase 1 (2.2-fold) in the posterior intestine of rainbow trout exposed to *Y. ruckeri* showed that the abundance of phagocytosis in the gut of rainbow trout results in enhanced intestinal defense against *Y. ruckeri*. Finally, these results suggest that *Y. ruckeri* triggers intestinal inflammation which enhances growth in the intestinal lumen and supports the invasion of the intestinal epithelium and mucosal macrophages. This macrophagic process promotes phagocytic activity in the gut to eliminate the bacteria and plays an important role in maintaining gut homeostasis and immune response [20].

In addition, caspase 6 and precerebellin-like protein were upregulated in the posterior intestine. Caspases are a family of proteases engaged in various important biological processes and play a significant role in the execution phase of the apoptotic death cascade [47]. Caspase-6 was found to be involved in apoptosis and immune response in puffer fish (*Takifugu obscurus*) against *Aeromonas hydrophila* [48]. The upregulation of caspase-6 (2.2-fold) in the gut mucosa of rainbow trout suggests that caspase-6 plays a role in the immune response and cell apoptosis against *Y. ruckeri* infection. However, another apoptosis related protein, programmed cell death protein 5 (PDCD5), was downregulated (−2.7- to 4.4-fold) in the posterior intestine as well. This indicates that PDCD5 may have an important role in the pathogenesis and development of the disease. However, the mechanisms underlying its apoptotic function are largely unknown in the fish. Precerebellin-like protein is a part of the acute phase response and has been characterized in rainbow trout [49]. The expression of precerebellin-like protein gene was upregulated (sevenfold) in the liver of rainbow trout in response to *Y. ruckeri* at 3 dpe [50]. This suggests that acute phase proteins act as a defensive agent against *Y. ruckeri* on the gut mucosal surface of rainbow trout. In support of this, the expression of some acute phase proteins such as serum amyloid protein and hepcidin was observed to be significantly increased in rainbow trout after *Y. ruckeri* challenge [50].

Evidently, calcium is engaged in signal transduction by acting as a second messenger [51]. In our study, we found two calcium binding proteins (calretinin and regucalcin) and seven signal transduction proteins (Ras-related protein Rab-1B, Rab-5C, Rab7-like, Rab-32-like, Rab-25-like, Rab7-like and ORAB-1) being upregulated. Calretinin is a calcium-binding protein found broadly distributed in the central nervous system and regucalcin is crucial for the regulation of Ca^2+^ ion homeostasis [52, 53]. The distribution of calretinin immunoreactivity in the developing olfactory system of the rainbow trout was observed by using an immunocytochemistry technique [54], and the expression of regucalcin gene was upregulated in rainbow trout at different temperatures and *Aeromonas salmonicida* challenges [55]. It could be speculated that calretinin and regucalcin execute their functions indirectly by controlling Ca^2+^ homeostasis in infected rainbow trout. Additionally, four calcium binding proteins [annexin A-13 (−4.1-fold), protein S100 (−6.6-fold), M-calpain (−4.0-fold), and calpain 2 catalytic subunit (−2.6-fold)] were downregulated. Annexins are a member of a multigene family of Ca^2+^ and phospholipid binding proteins [56]. S100 proteins take part in many cellular processes such as modulation of protein kinases and signal transduction pathways, maintenance of cell shape, regulation of calcium homoeostasis [57] and interaction with the parasite, *Tetracapsuloides bryosalmonae* [58]. However, calpains are calcium regulated proteases involved in many cellular functions and have been characterized in rainbow trout [59]. In conclusion, the presented results establish the significance of calcium binding proteins and their downregulation in the gut of rainbow trout in response to *Y. ruckeri*.

*Yersinia ruckeri* causes profound alterations in the nutritional and metabolic status of the fish [18, 60]. The interaction of a pathogen with intestinal mucosa leads to a variety of physiological responses aimed at adjusting to the new condition and triggers different processes in the gut epithelial cells [20]. Thus, we found upregulation of anabolic and catabolic proteins [fructose-1,6-bisphosphatase 1 (3.8-fold), beta-hexosaminidase (2.6-fold), alpha-*N*-acetylgalactosaminidase (4.1-fold), and long-chain specific acyl-CoA dehydrogenase (2.6-fold)], transport proteins [epididymal secretory protein E1 (3.9-fold), v-type proton ATPase subunit a (2.2-fold), and AP-1 complex subunit mu-2 (3.1-fold)]. The up-regulation of fructose-1,6-bisphosphatase and epididymal secretory protein E1 was observed in the spleen of rainbow trout in response to *Y. ruckeri* [30]. This suggests that these proteins are involved in the gut’s immune response and might result in physiological adaptations that contribute to extended longevity of fish during bacterial infection. Additionally, we found some downregulated proteins in the posterior intestine that are involved in gluconeogenesis [l-lactate dehydrogenase (−7.4-fold)], lipid transport [apolipoprotein A-I-1 (−2.2-fold), apolipoprotein A-II (−4.0-fold), and apolipoprotein C-I (−4.6-fold)], and translation [eukaryotic translation initiation factor 5A (−2.4-fold), 40S ribosomal protein S29 (−3.4-fold), and 40S ribosomal protein S12 (−2.2-fold)]. This suggests that fish might adjust their metabolism to channel the energy to the process of defense by decreasing glycogenesis, lipid metabolism, and translation, and increasing carbohydrate and protein catabolism during yersiniosis.

In conclusion, this study provides the first evidence illustrating the proteomic alteration of the intestinal mucosa of rainbow trout in response to *Y. ruckeri*, suggesting *Y. ruckeri* exerts a profound impact on posterior intestine mainly at 3 dpe. Intestinal endopeptidase, exopeptidase, and the proteins involved in antioxidant defense processes were upregulated upon *Y. ruckeri* exposure, while those involved in lipid metabolism, actin filament, and translation processes were downregulated. It is expected that this new information will exemplify further how *Y. ruckeri* influences the intestinal mucosa proteome of fish. The predicted lysosomal, metabolic, and focal adhesion pathways might be useful in understanding the gut defense mechanisms of rainbow trout and further research work in this direction. Utilization of these results may improve approaches for selection of disease-resistant rainbow trout broodstock and evaluation of prevention opportunities. Finally, the relationship among the intestinal bacterial pathogen and host protein responses will be explored to improve nutrient uptake, fish performance, and vaccine efficiency. Further studies are needed to address the functions of these intestinal proteins in immune response and protection against bacterial infection for both disease control and improved performance.

## Additional files

**Additional file 1.** **Ion library.** Text file based on IDA runs (independent data acquisition) of the intestine samples used for data interpretation of SWATH acquisitions obtained by PeakView 2.2 software (Sciex).

**Additional file 2.** **List of quantitative real-time PCR primers for confirmation of expression data.** PCR primers specific to the selected genes were designed using NCBI Primer BLAST software.

**Additional file 3.** **The details of total identified posterior intestine proteins of rainbow trout.** The number of proteins was identified at false discovery rate of 1% and minimum two peptides.

**Additional file 4.List of up or down-regulated posterior intestine proteins of rainbow trout in response to** ***Yersinia ruckeri*** **strains at 3 dpe.** Fold change was statistically analyzed in the posterior intestine of rainbow trout exposed to *Y. ruckeri* biotype 1 (CSF007-82) and biotype 2 (A7959–11) versus control posterior intestine rainbow trout samples (*n* = 3 per time point). * denotes statistically significant difference according to both ANOVA and post hoc Tukey’s HSD with FDR-adjusted *p*-value < 0.05 and fold change < −2 or > +2.

## Data Availability

Shotgun proteomics data generated during the current study have been deposited in the ProteomeXchange Consortium via the PRIDE partner repository [61] with the dataset identifiers PXD011087.

## Abbreviations

ERM: enteric redmouth disease
FDR: false discovery rate
IDA: information dependent data acquisition
LC–MS: liquid chromatography–mass spectrometry
Micro LC ESI QTOF MS/MS: microflow high-performance liquid chromatography electrospray-ionisation quadrupole time-of-flight tandem mass spectrometry
PCA: principal component analysis
SWATH: sequential window acquisition of all theoretical spectra
TOF: triple quadrupole time of flight
