# Supplementary material for: Modulation of posterior intestinal mucosal proteome in rainbow trout (Oncorhynchus mykiss) after Yersinia ruckeri infection
Source: Vet Res. 2019 Jul 17;50:54. doi: 10.1186/s13567-019-0673-8 (PMC6637484; doi:10.1186/s13567-019-0673-8)
Supplement: Supplementary file 2 — Additional file 2. List of quantitative real-time PCR primers for confirmation of expression data. PCR primers specific to the selected genes were designed using NCBI Primer BLAST software. [file 13567_2019_673_MOESM2_ESM.doc]

**Additional file 2 List of quantitative real-time PCR primers for confirmation of expression data.** PCR primers specific to the selected genes were designed using NCBI Primer BLAST software.

| Primer code | Sequence (5`-3`) | Amplicon size (bp) | Annealing temperature (°C) | NCBI accession no. |
| --- | --- | --- | --- | --- |
| Probable serine carboxypeptidase F | CGCTCTTTCTCACCCCCTAC | 192 | 57 | XM_021586185.1 |
| Probable serine carboxypeptidase R | GCAGGACTGGAGCAATCTCA |
| Cathepsin D F | CCTCAACAGGAACCCAGACT | 176 | 57 | NM_001124711.1 |
| Cathepsin D R | CACAGCCTCCCTTACACAGA |
| Caspase 6 F | GCTTACGACGACAAGATCGC | 180 | 57 | NM_001124271.1 |
| Caspase 6 R | CACCACCTCGTTGGTCTTCA |
| Lysozyme II F | GCTGTTGTTGTTCTCCTGCT | 129 | 57 | NM_001124716.1 |
| Lysozyme II R | GCAAACCCAGTTGGGCAG |
| Precerebellin 2F | GAGTCTGTTTTGGCTGTGGT | 131 | 57 | AF192969.2 |
| Precerebellin 2R | GCCTTCCCTGAATTCCCAAC |
| S100 F | GCCTTCTCTCCTGAAGGCTTCTAA | 162 | 58 | XM_021578278.1 |
| S100 R | GGAGCTCATTTCTTGGGGCA |
| Tubulin alpha F | CAATCAAGACCAAGCGCACC | 104 | 57 | NM_001141467.2 |
| Tubulin alpha R | CAGATCTCCTCCAGGGACCA |
| EF-1α F* | AGACAGCAAAAACGACCCCC | 167 | 57 | HF563594 |
| EF-1α R* | AACGACGGTCGATCTTCTCC |
| *Y. ruckeri* 16S rRNA F** | GCGAGGAGGAAGGGTTAAGTG | 70 | 60 | X75275 |
| *Y. ruckeri* 16S rRNA R** | GTTAGCCGGTGCTTCTTCTG |

*as published in Kumar et al. [38]

**as published in Raida and Buchmann [39]
